# A Rare Case of Ischial Tubercle Pressure Sore with Secondary Periperineal Necrotizing Fasciitis

**DOI:** 10.2174/0118715303385272250320042418

**Published:** 2025-04-04

**Authors:** Peiqi Wang, Yiyang Liu, Junhua Wang, Qiaofeng Guo, Xiang Wang

**Affiliations:** 1 Department of Surgery, Pan'an County Traditional Chinese Medicine Hospital, Jinhua 322300, China;; 2 Department of Orthopaedics, Tongde Hospital of Zhejiang Province, Hangzhou 310012, China;; 3 Nanjing Drum Tower Hospital Clinical College of Traditional Chinese and Western Medicine, Nanjing University of Chinese Medicine, Nanjing 210008, China;; 4 ICU, Pan'an County Traditional Chinese Medicine Hospital, Jinhua 322300, China

**Keywords:** Pressure ulcer, ischial tuberosity, perineum, necrotizing fasciitis, surgical debridement, antimicrobial therapy

## Abstract

**Background:**

Perineal necrotizing fasciitis, or Fournier's gangrene, is a rare but rapidly progressing condition characterized by fascial necrosis. It is a severe, potentially life-threatening infection requiring prompt diagnosis and standardized treatment to optimize patient outcomes.

**Case Presentation:**

A 48-year-old woman with poorly controlled type 2 diabetes developed necrotizing fasciitis of the right perineum secondary to an ischial tuberosity pressure ulcer. She had a prior spinal cord injury resulting in sensory dysfunction in the lower limbs, which masked significant pain. Management included surgical debridement, open wound care, antimicrobial therapy, and a free skin graft for wound closure.

**Conclusion:**

Effective treatment of necrotizing fasciitis relies on aggressive debridement and appropriate antimicrobial therapy. This case highlights the importance of early recognition and intervention to improve clinical diagnostic and management strategies.

## 1 INTRODUCTION

Necrotizing fasciitis is a rare but aggressive soft tissue infection with a rapid progression and high severity [1, 2]. Its annual incidence ranges from 0.3 to 15 cases per 100,000 people [3-5]. The infection originates in the fascia, swiftly spreading to subcutaneous fat, superficial and deep fascia and the skin. While it can develop anywhere in the body, the limbs, perineum, and abdomen are most commonly affected [6]. Necrotizing fasciitis in the perineal region, termed Fournier's gangrene [ 6 ] , accounts for approximately 21% of cases [ 7 ] , with diabetes being the primary risk factor [8]. The disease progresses rapidly, initially presenting with erythema, swelling, and pain before advancing to tissue liquefaction and necrosis. The lesion expands progressively, and systemic toxin absorption may result in bacteremia, sepsis, or even septic shock, posing a life-threatening risk. Early diagnosis and standardized treatment are critical to improving patient prognosis and survival.

## 2 CASE PRESENTATION

A 48-year-old woman presented with bilateral ischial tuberosity pressure ulcers persisting for 6 months, with recent exacerbation of the right-sided ulcer, perineal breakdown, and discharge over the previous 4 days. She reported no significant fever or pain in the right buttock or perineal region. Her husband noted that the necrotic area in the perineum had progressively enlarged, with increasing discharge and a worsening foul odor.

**Fig. (1):**
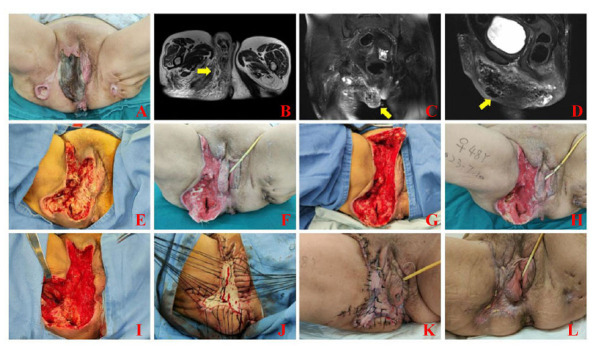
Case 1: A 48-year-old woman was hospitalized with bilateral ischial tuberosity pressure ulcers persisting for six months, which had worsened over four days, leading to right perineal tissue breakdown and purulent discharge. (**A**): Preoperative perineal view: A wound with a diameter of approximately 4 cm is present at the right ischial tuberosity. The right perineal skin appears necrotic and discolored, with purulent discharge. Magnetic Resonance (MR) images (axial (**B**), coronal (**C**), and sagittal (**D**) views) reveal a honeycomb-like pattern in the right perineal subcutaneous soft tissue, with necrosis, fluid accumulation, and gas pockets (yellow arrows). (**E**): Following the initial debridement, most of the necrotic tissue was excised, leaving only a minimal portion of marginally viable tissue. (**F**): After one week of open wound care, fresh granulation tissue is observed, with minimal purulent exudate. (**G**): The second debridement completely removed all necrotic and scar tissue. (**H**): Another week of open wound management resulted in healthy granulation tissue without purulence or necrosis. (**I**): After the third debridement, the wound bed shows good viability for grafting. (**J**): Full-thickness skin grafting, post-suture fixation. (**K**): Nine days postoperatively, the skin graft appears well-integrated, and swelling of the right major labia has resolved. (**L**): At the six-month follow-up, the graft on the right ischial tuberosity and perineum remains viable. Localized scar formation and mild contracture are noted. The groin skin has adhered well to deep tissues, with no residual cavity, and the labia majora has regained normal morphology.

Upon admission, the patient had a temperature of 36.9°C, pulse rate of 93 beats/min, respiratory rate of 18 breaths/min, and blood pressure of 94/58 mmHg. She exhibited diminished consciousness and bilateral ischial tuberosity pressure ulcers, with the right side being more severe and showing significant purulent exudate. The right perineal region was swollen, with necrotic skin extending to the labia majora and a foul-smelling purulent discharge (Fig. **1A**). Eight years prior, she had undergone thoracic spine tumor surgery, resulting in persistent sensory and motor deficits below the umbilicus, as well as urinary and fecal dysfunction. She had been diagnosed with diabetes for the same duration but had neither adhered to regular medication nor monitored her blood glucose consistently. At admission, her fasting blood glucose was 12.44 mmol/L, glycated hemoglobin was 9.5%, and total glycated hemoglobin was 12.3%. Laboratory findings included white blood cell count 16.6×10^9^/L, neutrophils 86.0%, lymphocytes 9.7%, red blood cell count 2.37×10^12^/L, hemoglobin 64 g/L, ultra-sensitive C-reactive protein 249.0 mg/L, erythrocyte sedimentation rate 162mm/h, procalcitonin 1.652 ng/mL, albumin 23.4 g/L, globulin 38.6 g/L, total protein 62.0 g/L, and an albumin-to-globulin ratio of 0.6 Wound cultures identified *Proteus mirabilis*, resistant to cephalosporins and carbapenems but sensitive to piperacillin/tazobactam and levofloxacin. Mild coagulation abnormalities were also noted. The admission diagnoses included septic shock, right inguinal and perineal necrotizing fasciitis, bilateral ischial tuberosity pressure ulcers, diabetes mellitus, spinal cord injury, anemia, and hypoalbuminemia. The patient received immediate treatment with Ringer's solution, hydroxyethyl starch for fluid resuscitation, and piperacillin/sulbactam sodium for infection control. Homologous red blood cells and plasma were administered to address anemia, hypoalbuminemia, and coagulation dysfunction. Concurrently, insulin therapy was initiated for glycemic control. Magnetic Resonance Imaging (MRI) findings revealed extensive tissue necrosis with fluid and gas accumulation in the subcutaneous and fascial layers, extending from the right buttock to the perineum. A honeycomb-like pattern was observed in localized tissue (Figs. **1B**-**D**). On the second day, under general anesthesia, debridement was performed for bilateral ischial tuberosity pressure ulcers and necrotizing fasciitis of the right perineum, followed by open drainage. Intraoperatively, necrotic tissue in the right perineal region was excised. The wound extended anteriorly to approximately 3 cm above the pubic symphysis, posteriorly to the anal level, laterally to the ischial tuberosity, and medially to the labia majora. The fascial layer and overlying tissue were completely necrotic, though the deep gluteal and proximal adductor muscles remained uninvolved. Some tissue with potential viability was preserved (Fig. **1E**). Postoperatively, wound dressings were changed regularly, and debridement was repeated after one week. The wound was largely clean at that time, with minimal necrotic tissue and no purulent exudate. Edema in the labia majora had partially resolved (Fig. **1F**). The second debridement revealed a subcutaneous cavity extending towards the pubic symphysis, measuring approximately 5 cm deep and 4 cm wide (Fig. **1G**). After an additional week of open wound care, fresh granulation tissue had formed, and the edema had further subsided (Fig. **1H**). At this stage, fasting blood glucose was 6.48 mmol/L. Laboratory findings included a white blood cell count of 8.0×10^9^/L (neutrophils: 62.4%, lymphocytes: 29.8%), red blood cell count of 3.29×10^12^/L, and hemoglobin of 96 g/L. Ultra-sensitive C-reactive protein was 11.40 mg/L, erythrocyte sedimentation rate was 93 mm/h, and procalcitonin was 0.073 ng/mL. Serum albumin was 29.5 g/L, globulin 34.5 g/L, total protein 64.0 g/L, with an albumin-to-globulin ratio of 0.9 Wound bacterial cultures were negative. During the third debridement, the right ischial tuberosity pressure sore was fully opened (Fig. **1I**). Skin flaps surrounding the wound were sutured to the deep tissue, closing the local defect. Similarly, the lower abdominal and inguinal wounds were sutured to eliminate dead space. The residual skin defect was covered with a full-thickness graft harvested from the lower abdomen, which was evenly packed and compressed (Fig. **1J**). Bolster dressings were removed nine days later, confirming full graft integration and successful wound healing (Fig. **1K**). At the six-month follow-up, bilateral pressure ulcers remained healed without recurrence. The inguinal-to-perineal wound had healed well, with a mildly concave local appearance. Minimal scar contracture was present, and the labia majora had largely regained normal morphology (Fig. **1L**).

## 3 DISCUSSION

Necrotizing fasciitis is a rapidly progressing, life-threatening soft tissue infection that primarily affects the deep and superficial fascia and subcutaneous tissue. Although its incidence is relatively low, the condition is severe, with a mortality rate exceeding 20% [4, 9]. When necrotizing fasciitis involves the perineal region, it is classified as Fournier's gangrene, a distinct subtype first described in 1883 [6]. This infection affects the perineal and/or perianal skin and subcutaneous tissues, with reported mortality rates ranging from 30% to 35% [10, 11]. Without timely intervention, mortality can escalate to an alarming 67% [12].

The disease is significantly more prevalent in men, with reported male-to-female ratios in the literature varying from 2.5:1 to 42:1 [12-14]. Although less common in women, infections in this group tend to extend from the groin to the lower abdominal wall [14]. The primary bacterial pathogens associated with Fournier’s gangrene include *Escherichia coli, Pseudomonas aeruginosa, Enterococcus faecalis*, and *Staphylococcus aureus* [14]. Current guidelines from the Infectious Diseases Society of America (IDSA) recommend an initial empirical antibiotic regimen combining vancomycin or linezolid with piperacillin-tazobactam, carbapenems, or ceftriaxone-metronidazole. Subsequent treatment should be tailored based on Gram staining findings, culture results, and antimicrobial susceptibility testing of infected soft tissue [15].

Necrotizing fasciitis is primarily associated with risk factors such as diabetes, obesity, immunosuppression, alcohol abuse, renal failure, peripheral vascular disease, liver disease, and malignancies, although some idiopathic cases exist [14, 16]. Diabetes is the most common predisposing factor, responsible for approximately 45-57% of cases [17-19]. Among patients with Fournier's gangrene, diabetic prevalence reaches 76.9% [ 14 ] , and glycemic control is directly linked to disease severity and prognosis [14]. Once necrotizing fasciitis develops, infection-induced tissue necrosis can further elevate blood glucose levels, creating a vicious cycle. Therefore, strict glycemic management is crucial for reducing disease occurrence. Early-stage necrotizing fasciitis typically presents with erythema, swelling, and pain, progressing rapidly if untreated. In patients with diabetes, vascular complications increase the risk of gangrene [20]. Moreover, skin injuries facilitate bacterial colonization and tissue invasion, with reports indicating that nearly half of affected patients have a history of skin trauma [3].

In this case, four key factors contributed to disease progression: 1. A chronic sacral pressure ulcer served as the primary external trigger for perineal necrotizing fasciitis; 2. Persistent hyperglycemia was the most significant internal factor, accelerating disease onset and progression; 3. Spinal cord injury caused sensory impairment, reducing pain perception, and delaying diagnosis and treatment; 4. Due to local anatomical factors, the infection extended beyond the perineal and perianal regions to involve the inguinal and abdominal wall subcutaneous tissues [14].

The standard approach to necrotizing fasciitis management includes early diagnosis, prompt debridement, broad-spectrum antibiotic therapy, aggressive fluid resuscitation, frequent reassessment, and comprehensive nutritional support [14, 18, 19]. Surgical debridement remains the cornerstone of treatment. Early, thorough debridement significantly reduces mortality risk [9, 13, 16]. All infected and devitalized tissues must be excised intraoperatively, with repeated irrigation using hydrogen peroxide and copious saline. Special attention is required to assess deep tissue necrosis, which frequently extends beyond visible skin lesions [16]. Debridement aims to expose all healthy deep and superficial tissues [16], ensuring complete removal of all necrotic material. Due to the wound’s anatomical location, commonly used techniques such as closed negative pressure drainage and bone cement coverage were not feasible. Therefore, postoperative management involved open dressing changes. Granulation tissue formation was robust following two debridement procedures and ongoing open wound care. The surgical team initially considered a gracilis muscle flap for sacral pressure ulcer repair and perineal reconstruction. However, due to the patient's personal preferences, a full-thickness skin graft was performed instead. The wound healed well postoperatively, though localized scarring and contour loss were noted compared to the unaffected side.

For most post-debridement wounds of necrotizing fasciitis, primary closure is rarely an option. Free skin grafting and flap transplantation are the primary reconstructive techniques. Free skin grafting is straightforward, with minimal surgical trauma, high graft survival rates, and lower short-term risks. However, long-term concerns include scar contracture and poor durability [14, 21]. These challenges are particularly relevant where grafting is performed over the ischial tuberosity, where sustained pressure increases the risk of recurrent ulceration and pressure sores. Various flap repair techniques are available for perineal wounds, with the “petal flap” approach [ 22 ] gaining widespread recognition. Moreover, muscle and musculocutaneous flaps from the medial and posterior thigh regions offer effective soft tissue reconstruction for different perineal defects.

## CONCLUSION

Necrotizing fasciitis is characterized by distinct risk factors, with diabetes and skin trauma being the most significant internal and external contributors. Early diagnosis and intervention play a crucial role in improving prognosis. The foundation of treatment lies in thorough debridement and aggressive infection control, which create the conditions for successful wound closure. This report presents a rare case of perineal necrotizing fasciitis in a female patient secondary to an ischial tuberosity pressure ulcer. This study aimed to enhance diagnostic and treatment strategies for similar cases by documenting the clinical course and therapeutic approach in detail.

## Data Availability

The datasets generated and analyzed during this study are available from the corresponding author upon reasonable request.

## LIST OF ABBREVIATIONS

MRI: Magnetic Resonance Imaging
IDSA: Infectious Diseases Society America

## STANDARDS OF REPORTING

CARE guidelines were followed.
